# Conditions for the existence of isolated backbone curves

**DOI:** 10.1098/rspa.2019.0374

**Published:** 2019-12-04

**Authors:** Dongxiao Hong, Thomas L. Hill, Simon A. Neild

**Affiliations:** Department of Mechanical Engineering, University of Bristol, Bristol BS8 1TR, UK

**Keywords:** structural dynamics, nonlinear normal mode, backbone curves, isola, perturbation analysis

## Abstract

Isolated backbone curves represent significant dynamic responses of nonlinear systems; however, as they are disconnected from the primary responses, they are challenging to predict and compute. To explore the conditions for the existence of isolated backbone curves, a generalized two-mode system, which is representative of two extensively studied examples, is used. A symmetric two-mass oscillator is initially studied and, as has been previously observed, this exhibits a perfect bifurcation between its backbone curves. As this symmetry is broken, the bifurcation splits to form an isolated backbone curve. Here, it is demonstrated that this perfect bifurcation, indicative of a symmetric structure, may be preserved when the symmetry is broken under certain conditions; these are derived analytically. With the symmetry broken, the second example—a single-mode nonlinear structure with a nonlinear tuned mass damper—is considered. The evolution of the system's backbone curves is investigated in nonlinear parameter space. It is found that this space can be divided into several regions, within which the backbone curves share similar topological features, defining the conditions for the existence of isolated backbone curves. This allows these features to be more easily accounted for, or eliminated, when designing nonlinear systems.

## 1. Introduction

Over recent decades, demand on the performance of engineering structures has been continually growing. Meeting this demand often requires extending the performance envelope of structures to regions where nonlinearity must be accounted for. This results in complex nonlinear dynamic phenomena, such as bifurcations, internal resonances and multiple solutions [1–6]. Despite the challenges these phenomena have posed to analysis and design, recent studies seek to exploit, rather than avoid, the nonlinear behaviours. Application areas include vibration absorbers and energy harvesting [7–13]. Among these applications, the nonlinear tuned mass damper (NLTMD) has been extensively studied in the literature with its advantageous performance demonstrated [14–17].

Den Hartog [18] proposed a widely adopted method to optimize the parameters of a linear tuned mass damper (LTMD). Later, a nonlinear generalization of Den Hartog's equal-peak method for an NLTMD was established in [16], where the performance between the NLTMD and the LTMD was compared. Gatti [17] highlights that the main advantage of introducing nonlinearity is the improvement of the bandwidth of the device. An NLTMD has, for example, been used to control a supercritical Hopf-bifurcation of aerofoil flutter in [19], and to control limit cycle oscillations of mechanical systems in [20].

Besides these favourable properties, the use of an NLTMD may bring some undesirable dynamic phenomena, such as isolas, i.e. forced responses that are isolated from the primary response branches. Due to this feature, the existence of isolas can be difficult to determine; furthermore, these isolated solutions may represent significant, high-amplitude responses [16,21–23]. An early study of isolas in engineering systems was carried out by Abramson [24] in 1955. Extensive work since this has focused on the mechanism for their creation, such as discontinuity [25,26], internal resonances [27] and symmetry breaking [28,29].

Numerous approaches have been used to detect and trace isolas. One numerical method is continuation which uses special points, such as fold bifurcations and extremum points, to trace the evolution of isolas by varying specific parameters [30,31]. In combination with continuation methods, singularity theory can be used to provide complementary information in the prediction and identification of isolas [32–34]. Methods based on continuation can efficiently find isolas; however, they require a good understanding of the system, and its responses, to select the appropriate continuation parameters. Another numerical method is global analysis, which may detect an isola by finding initial conditions which are within the basin of attraction of that isola [35]. This approach requires a large number of simulations of initial conditions, making it computationally expensive. In addition to these methods, experimental techniques are also used to detect isolas; for example, using control-based continuation, an isola is detected for a nonlinear beam structure in [36].

An alternative approach to considering forced responses is to analyse the underlying backbone curves of the unforced, undamped system. Backbone curves, which are also known as nonlinear normal modes (NNMs), are also widely used in nonlinear modal analysis, reduced-order modelling and localization analysis (e.g. [5,6,37]). Backbone curves can be related to the forced responses of a system (including those that lie on isolas) using energy balance analysis [29,30,38]. This involves finding the points in the forced responses that lie on the backbone curves, such that the forcing energy in matches the damping energy loss. This approach reduces the isola-finding problem to an analytical and computationally simpler one; however, it requires that the backbone curves are known.

To complicate matters, while less studied than the forced counterpart, backbone curves themselves can also be isolated.^1^ Isolated backbone curves have been demonstrated for a simple, near-symmetric two-mass oscillator [38], and it was shown that this isolated curve emerged due to the breaking of symmetry. Recently, an isolated backbone curve has also been measured experimentally for a cross-beam system using a nonlinear force appropriation technique [39], and again shown to evolve from the symmetry breaking in the system. Without *a priori* knowledge of such isolated backbone curves, any isolated forced responses that are associated with them may go undetected. While the aforementioned systems exhibit isolated backbone curves during symmetry-breaking, in practice, some systems cannot be symmetric, e.g. a grounded structure with an ungrounded NLTMD attached, and the existence of isolas can have a significant impact on their performance, as discussed above. A general methodology, describing the relationship between the symmetry of the system and the evolution of isolated backbone curves, has not yet been fully explored. To understand this relationship, a more general case needs to be considered. Such insights into this relationship would ensure that isolated backbone curves can be reliably predicted when designing nonlinear devices and structures.

This paper presents a technique to determine the existence of isolated backbone curves for a two-mode system with cubic nonlinearities and a 1 : 1 internal resonance. This is motivated by the fact that much of the current literature on modal interactions consider systems where just two modes interact. The general two-mode model is related to two specific example systems: an in-line two-mass oscillator is used to explore the relationship between symmetry breaking and isolated backbone curves; the second motivating example is an NLTMD attached to a single-mode nonlinear structure. Exploiting the method developed for the general two-mode case, the evolution of isolated backbone curves in nonlinear parameter space is identified for the NLTMD system. To this end, the rest of this paper is organized as follows.

Section 2 firstly revisits the backbone curves of a two-mode symmetric system, which has been considered extensively (e.g. [5,29,37,38,40,41]). This system exhibits two single-mode backbone curves with one perfect bifurcation leading to mixed-mode backbone curves. By breaking the symmetry of this system, the perfect bifurcation splits to form an isolated backbone curve. It is then shown that, similarly to a symmetric system, an asymmetric system may also exhibit single-mode backbone curves with a perfect bifurcation. The mechanisms by which symmetry breaking affects the modal equations are then explored. Section 3 considers a nonlinear structure with an NLTMD and, using the insights from §2, derives the analytical parameter relationships to obtain two single-mode backbone curves with one perfect bifurcation. Perturbing the linear and nonlinear parameters from these relationships, the evolution of backbone curves in nonlinear parameter space is then discussed, and the emergence and evolution of isolated backbone curve addressed. Focusing on the system with hardening springs, discriminant analysis is used in §4 to find the analytical conditions under which the isolated backbone curve may be removed, i.e. shifted to infinite frequency and amplitude. Analytical expressions found in §§3 and 4 serve as boundaries, distinguishing topological features of backbone curves in nonlinear parameter space, and defining conditions for the existence of isolated backbone curves. Lastly, this paper is closed with conclusion in §5.

## 2. Breaking the symmetry of a nonlinear 2-d.f. oscillator

In this section, a general two-mode^2^ conservative system, with cubic nonlinearities, is firstly considered, before considering a specific two-mode system. The Lagrangian of this general system may be written

2.1 $$L=\frac{1}{2}{\dot{q}}_{1}^{2}+\frac{1}{2}{\dot{q}}_{2}^{2}-\frac{1}{2}\omega_{n1}^{2}q_{1}^{2}-\frac{1}{2}\omega_{n2}^{2}q_{2}^{2}-\frac{1}{4}\Psi_{4}q_{1}^{4}-\Psi_{1}q_{1}^{3}q_{2}-\frac{1}{2}\Psi_{3}q_{1}^{2}q_{2}^{2}-\Psi_{2}q_{1}q_{2}^{3}-\frac{1}{4}\Psi_{5}q_{2}^{4},$$

where q_i_, ${\dot{q}}_{i}$ and *ω*_ni_ are the ith linear modal displacement, velocity and natural frequency respectively, and *Ψ*_1_, …, *Ψ*_5_ are the nonlinear coefficients. Note that the nonlinear coefficients are defined in this order for simplicity in later sections. Applying the Euler–Lagrange equation then leads to the following equations of motion:

2.2*a* $${\ddot{q}}_{1}+\omega_{n1}^{2}q_{1}+\Psi_{4}q_{1}^{3}+3\Psi_{1}q_{1}^{2}q_{2}+\Psi_{3}q_{1}q_{2}^{2}+\Psi_{2}q_{2}^{3}=0$$

and

2.2*b* $${\ddot{q}}_{2}+\omega_{n2}^{2}q_{2}+\Psi_{1}q_{1}^{3}+\Psi_{3}q_{1}^{2}q_{2}+3\Psi_{2}q_{1}q_{2}^{2}+\Psi_{5}q_{2}^{3}=0.$$

Note that the use of the Lagrangian in deriving these expressions restricts the number of nonlinear parameters to five, while ensuring that the equations of motion remain conservative [42,43].

From equation (2.2b), when the coefficient of q^3^_1_ equals 0 (i.e. when *Ψ*_1_ = 0), q_2_ = 0 is a solution (although coupling between the two modes still exists via other terms). Substituting this into equation (2.2a) gives the single-mode solution, representing a single-mode backbone curve, or NNM branch, which consists of only the first linear modal coordinate, q_1_, by solving

2.3 $${\ddot{q}}_{1}+\omega_{n1}^{2}q_{1}+\Psi_{4}q_{1}^{3}=0.$$

Note that *Ψ*_1_ = 0 is the special case which results in this single-mode backbone, a solution which cannot exist when *Ψ*_1_≠0. In addition to this single-mode solution, backbone curves containing contributions from both linear modes, i.e. mixed-mode backbone curves, can also be found when *Ψ*_1_ = 0. Likewise, when the coefficient of q^3^_2_ in equation (2.2a), *Ψ*_2_, equals 0, one can find the single-mode solution that consists of only the second linear modal coordinate, q_2_, from

2.4 $${\ddot{q}}_{2}+\omega_{n2}^{2}q_{2}+\Psi_{5}q_{2}^{3}=0.$$

Otherwise, when both *Ψ*_1_≠0 and *Ψ*_2_≠0, this system only has mixed-mode backbone curves. To find the backbone curves of the general two-mode system, the harmonic balance technique^3^ is used, firstly by assuming that the modal displacements may be written as

2.5 $$q_{i}\approx u_{i}=U_{i}\cos\left(\omega_{ri}t-\theta_{i}\right),$$

where u_i_ represents the fundamental response of q_i_, and where U_i_, *ω*_ri_ and *θ*_i_ are amplitude, response frequency and phase of u_i_, respectively. Note that this trigonometric solution is equivalent to the exponential form used in [38,41]. It is further assumed that the fundamental frequencies of the two modes are equal, i.e. *ω*_r1_ = *ω*_r2_ = *Ω*, hence, the response frequency ratio is 1 : 1. With the substitution of expressions (2.5) into the equations of motion (2b), and the non-resonant terms removed, one can obtain the time-independent solutions from

2.6*a* $$\begin{array}{rl} & 4\left(\omega_{n1}^{2}-\Omega^{2}\right)U_{1}+3\Psi_{4}U_{1}^{3}+\Psi_{3}U_{1}U_{2}^{2}\left[1+2\cos^{2}\left(\theta_{d}\right)\right]+3\left(\Psi_{2}U_{2}^{3}+3\Psi_{1}U_{1}^{2}U_{2}\right)\cos\left(\theta_{d}\right)=0, \end{array}$$

2.6*b* $$\begin{array}{rl} & 4\left(\omega_{n2}^{2}-\Omega^{2}\right)U_{2}+3\Psi_{5}U_{2}^{3}+\Psi_{3}U_{1}^{2}U_{2}\left[1+2\cos^{2}\left(\theta_{d}\right)\right]+3\left(\Psi_{1}U_{1}^{3}+3\Psi_{2}U_{1}U_{2}^{2}\right)\cos\left(\theta_{d}\right)=0 \end{array}$$

2.6*c* $$\begin{array}{rl} \mathrm{and}\; & \left[2\Psi_{3}U_{1}U_{2}\cos\left(\theta_{d}\right)+3\Psi_{1}U_{1}^{2}+3\Psi_{2}U_{2}^{2}\right]\sin\left(\theta_{d}\right)=0, \end{array}$$

where *θ*_d_ = *θ*_1_ − *θ*_2_. These equations can then be used to compute the backbone curves of the general two-mode system.

### (a) The backbone curves of a symmetric two-mass oscillator

**Figure 1.**
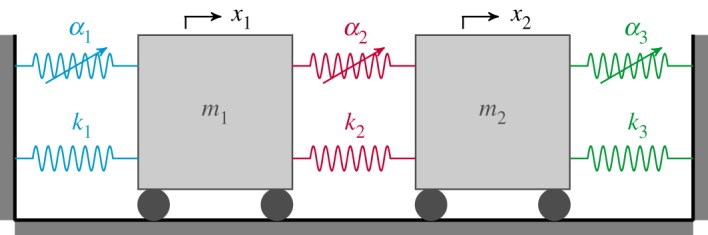
A schematic diagram of a two-mode system in the form of a two-mass oscillator. Two masses, with mass values m_1_ and m_2_, have displacements x_1_ and x_2_, respectively, while linear and nonlinear cubic springs have coefficients k_i_ and *α*_i_, respectively, where i = 1, 2, 3. (Online version in colour.)

To demonstrate the effect of symmetry breaking, a specific two-mode system, the two-mass oscillator shown schematically in figure 1, is now used. The system consists of two masses with mass values m_1_ and m_2_, and displacements x_1_ and x_2_, respectively. These masses are grounded via two linear springs, with coefficients k_1_ and k_3_, respectively, and are connected by another linear spring with coefficient k_2_. This system also contains three nonlinear cubic springs with coefficients *α*_1_, *α*_2_ and *α*_3_, as shown in figure 1. The backbone curves of this system can be computed from equations (2.6), using the relationship between the nonlinear modal coefficients, *Ψ*_i_, and the physical parameters of the model, as derived in appendix A.

**Figure 2.**
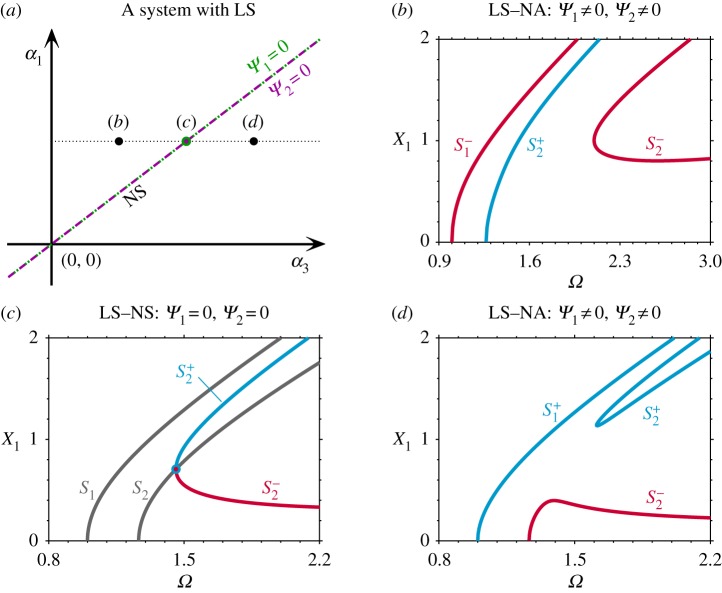
The effect of breaking the nonlinear symmetry (NS), i.e. breaking the condition *α*_1_ = *α*_3_, for a system with linear symmetry (LS), i.e. m_1_ = m_2_ and k_1_ = k_3_. (*a*) The nonlinear parameter space, *α*_1_ against *α*_3_, for the system with LS when m_1_ = m_2_ = 1, k_1_ = k_3_ = 1, k_2_ = 0.3 and *α*_2_ = 0.05. The *α*_1_ and *α*_3_ values that lead to *Ψ*_1_ = 0 and *Ψ*_2_ = 0 are shown as a green dotted line and a purple dashed line, respectively. (*b*) Backbone curves for a system with linear symmetry and nonlinear asymmetry (NA) when *α*_1_ = 1, *α*_3_ = 0.5 (represented by a black dot labelled (*b*) in panel (*a*)). (*c*) Backbone curves for an LS–NS system with *α*_1_ = 1, *α*_3_ = 1, where the solid point represents the perfect bifurcation (denoted by a solid dot labelled (*c*) in panel (*a*)). (*d*) Backbone curves for an LS-NA system with *α*_1_ = 1, *α*_3_ = 1.5 (represented by a black dot labelled (*d*) in panel (*a*)). Panels (*b*)–(*d*) are shown in the projection of the response frequency, *Ω*, against the amplitude of the first mass, X_1_. (Online version in colour.)

Here, the symmetry of the system is divided into two parts: linear symmetry (LS), where both m_1_ = m_2_ and k_1_ = k_3_; and nonlinear symmetry (NS), where *α*_1_ = *α*_3_. Note that the LS–NS case leads to *Ψ*_1_ = 0 and *Ψ*_2_ = 0, as shown in appendix A—see equation (A 9) with *α*_1_ = *α*_3_. As previously discussed, this leads to single-mode solutions. The backbone curves of the system with both LS and NS have been investigated in detail in [29,38,41], and an example is illustrated in figure 2*c*. The single-mode backbone curves S_1_ and S_2_ consist of only the first and second modal coordinates, respectively; while S^+^_2_ and S^−^_2_ represent mixed-mode backbone curves containing both linear modal coordinates. The subscripts of S^+^_2_ and S^−^_2_ indicate the backbone curve from which they bifurcate (i.e. from S_2_ in this case), and the superscripts^+^ and^−^ denote in-phase and anti-phase responses between the fundamental components of the linear modal coordinates, respectively. For details of how these backbone curves have been computed, using equations (2.6), see [29,38,41].

Here, we introduce the concept of *dynamic symmetry* to describe backbone curves with the features of an LS–NS system, specifically characterized by the following features:

(i) two single-mode backbone curves, S_1_ and S_2_.
(ii) two mixed-mode backbone curves; either S^±^_2_, emerging from a perfect bifurcation on S_2_, or S^±^_1_, emerging from a perfect bifurcation on S_1_.

### (b) Breaking **either** the nonlinear **or** the linear parameter symmetry

Figure 2*a* represents the nonlinear parameter space of *α*_1_ against *α*_3_—i.e. the parameters of the two nonlinear grounding springs—for the case where the system has LS. The nonlinear parameters leading to *Ψ*_1_ = 0 and *Ψ*_2_ = 0 are denoted by green dotted and purple dashed lines, respectively. In this case, i.e. for LS, these lines correspond to NS. In other words, for this simple system, both LS and NS will always lead to *Ψ*_1_ = *Ψ*_2_ = 0, hence resulting in single-mode backbone curves which is one of the conditions for dynamic symmetry. For nonlinear parameter combinations with nonlinear asymmetry (NA), i.e. *α*_1_≠*α*_3_, both *Ψ*_1_≠0 and *Ψ*_2_≠0, as indicated by the dots labelled (*b*) and (*d*) in figure 2*a*. This symmetry breaking turns the single-mode backbone curves into mixed-mode ones, breaks the perfect bifurcation, and generates an isolated backbone curve. As shown in figure 2*b*,*d* (corresponding to parameters labelled (*b*) and (*d*) in figure 2*a*), an isolated backbone curve emerges from two primary mixed-mode backbone curves, as observed in [29]. As proven in appendix A, a system with LS and NA cannot exhibit dynamic symmetry as in this case *Ψ*_1_≠0 and *Ψ*_2_≠0.

**Figure 3.**
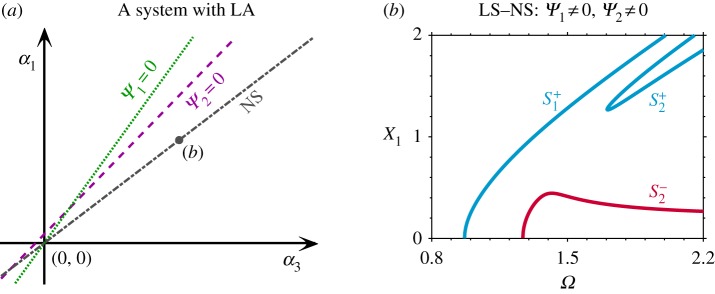
The effect of breaking the linear symmetry (LS) for a system with nonlinear symmetry (NS). (*a*) The nonlinear parameter space for the system with linear asymmetry (LA) when m_1_ = 1, m_2_ = 0.8, k_1_ = 1, k_3_ = 0.7, k_2_ = 0.3 and *α*_2_ = 0.05. The *α*_1_ and *α*_3_ values that lead to *Ψ*_1_ = 0 and *Ψ*_2_ = 0 are shown as a green dotted line and a purple dashed line, respectively, and parameters leading to NS are shown as a dash-dotted grey line. (*b*) Backbone curves for an LA–NS system with *α*_1_ = 1, *α*_3_ = 1 (represented by a grey dot labelled (*b*) in panel (*a*)) in the projection of the response frequency, *Ω*, against the amplitude of the first mass, X_1_. (Online version in colour.)

Similarly, breaking the LS, while retaining the NS, can also break the dynamic symmetry. With the breaking of the LS (m_2_ = 0.8m_1_ and k_3_ = 0.7k_1_), the orientations of *Ψ*_1_ = 0 and *Ψ*_2_ = 0 are changed, and are no longer overlapping, as shown in figure 3*a*. If the NS is retained, i.e. *α*_1_ = *α*_3_ (depicted by the grey line in figure 3*a*), the backbone curves, depicted in figure 3*b*, are similar in form to the ones for the LS–NA system in figure 2*d*, i.e. one isolated backbone curve between two primary mixed-mode backbone curves. The LA–NS system considered here cannot have dynamic symmetry since the intersection of *Ψ*_1_ = 0 and *Ψ*_2_ = 0, where one can find two single-mode backbone curves, is not on the line representing *α*_1_ = *α*_3_ (i.e. the point at which the green and purple lines in figure 3*a* cross does not correspond to the grey line).

### (c) Breaking **both** the linear **and** nonlinear parameter symmetry

**Figure 4.**
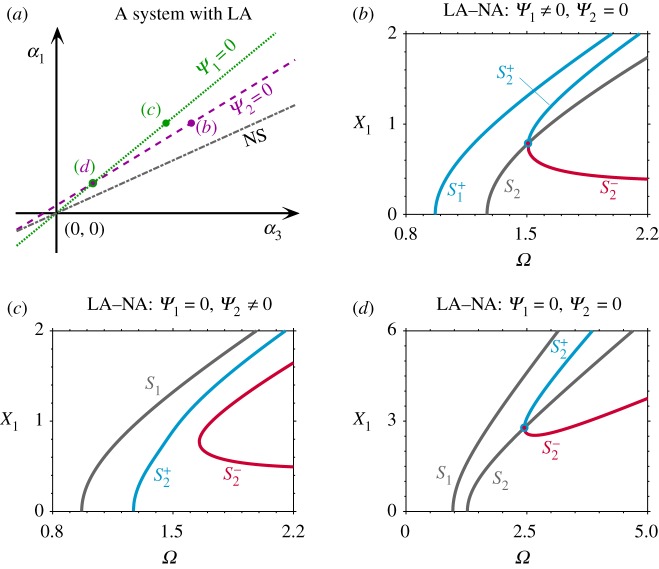
Obtaining dynamic symmetry for an LA–NA system. (*a*) The nonlinear parameter space for a system with LA (linear parameters and *α*_2_ are equal to those considered in figure 3). The *α*_1_ and *α*_3_ values that lead to *Ψ*_1_ = 0 and *Ψ*_2_ = 0 are shown as a green-dotted and a purple-dashed lines, respectively, and parameters leading to NS are shown as a dash-dotted grey line. (*b*) Backbone curves for an LA–NA system with *α*_1_ = 1, *α*_3_ ≈ 0.6785 (represented by a purple dot labelled (*b*) in panel (*a*)). (*c*) Backbone curves for an LA–NA system with *α*_1_ = 1, *α*_3_ ≈ 0.5510 (denoted by a green dot labelled (*c*) in panel (*a*)). (*d*) Backbone curves for an LA–NA system with *α*_1_ ≈ 0.3333, *α*_3_ ≈ 0.1833 (represented by a solid dot labelled (*d*) in panel (*a*)). (Online version in colour.)

Following from the LA–NS system considered in §2b, the NS is also broken to investigate the backbone curves of an LA–NA system. Figure 4*b* shows the backbone curves for the case where *α*_3_ is reduced from the NS-case to the point where *Ψ*_2_ = 0, marked as a purple dot labelled (*b*) in figure 4*a*. As expected, this leads to a single-mode backbone curve S_2_; however, as *Ψ*_1_≠0, the first primary backbone curve, S^+^_1_, contains a component of the second mode with in-phase modal coordinates. As such, this is not a dynamically symmetric case, despite sharing some characteristics, such as the backbone curves S^+^_2_ and S^−^_2_ which bifurcate off S_2_. Further reducing *α*_3_ leads to the point where *Ψ*_1_ = 0, shown as a green dot labelled (*c*) in figure 4*a*, whose backbone curves are shown in figure 4*c*. These exhibit a single-mode backbone curve S_1_ (as predicted by the *Ψ*_1_ = 0 condition) but with a primary and an isolated backbone curve, S^+^_2_ and S^−^_2_. As shown in figure 4*a*, *Ψ*_1_ = *Ψ*_2_ = 0 may still be satisfied for this case if *α*_1_ and *α*_3_ are on the intersection of *Ψ*_1_ = 0 and *Ψ*_2_ = 0, i.e. the point labelled (*d*) in figure 4*a*. Dynamic symmetry can therefore be obtained for such an LA–NA system, as can be seen from the backbone curves in figure 4*d*.

As previously discussed, the concept of dynamic symmetry is defined as having similar characteristics to an LS–NS system; nonetheless, such behaviour can be observed in an LA–NA system, if parameters are appropriately selected. This means that an LA–NA system can exhibit the same dynamic characteristics as a fully symmetric system. Furthermore, defining conditions for the existence of single-mode solutions, expressions *Ψ*_1_ = 0 and *Ψ*_2_ = 0 also serve as boundaries, which divide the nonlinear parameter space into several regions, within which the backbone curves share similar topological features. These regions, in the nonlinear parameter space, allow the changes in the fundamental dynamic behaviours to be identified and predicted.

## 3. Backbone curves for an NLTMD-inspired two-mode system

**Figure 5.**
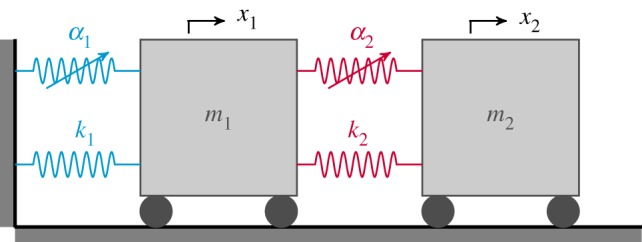
A schematic diagram of a two-mode asymmetric system, representative of a nonlinear structure with an NLTMD. (Online version in colour.)

In this section, a two-mode asymmetric system, depicted in figure 5, is considered. This system is equivalent to that shown in figure 1, but with the springs grounding the second mass removed. This system is representative of a nonlinear structure (first mass) with an NLTMD (second mass) attached.

Isolated backbone curves represent particularly undesirable features in an NLTMD device [16,21,23], due to the difficulty of predicting them, and their potential to represent high-amplitude dynamic responses. To further understand their features, in this section the parameter conditions required for dynamic symmetry are first found. However, in practice, the optimized linear parameters of an NLTMD cannot usually satisfy such conditions. The evolution of backbone curves in nonlinear parameter space, for an optimized set of linear parameters, is then investigated and used to determine the conditions for the existence of isolated backbone curves.

### (a) Parameter conditions required for dynamic symmetry

As discussed in §2, an LA–NA system can exhibit dynamic symmetry if the parameters are selected appropriately. One feature of dynamic symmetry is having two single-mode solutions, S_1_ and S_2_, which requires that both *Ψ*_1_ = 0 and *Ψ*_2_ = 0 in the equations of motion (2b). The expressions of *Ψ*_1_ and *Ψ*_2_ for the NLTMD system are given in equations (A 5), and may be written in matrix form as

3.1 $$\left(\begin{array}{c} \Psi_{1}\\ \Psi_{2} \end{array}\right)=\left[\begin{array}{cc} \phi_{11}^{3}\phi_{12} & {\left(\phi_{11}-\phi_{21}\right)}^{3}\left(\phi_{12}-\phi_{22}\right)\\ \phi_{11}\phi_{12}^{3} & \left(\phi_{11}-\phi_{21}\right){\left(\phi_{12}-\phi_{22}\right)}^{3} \end{array}\right]\left(\begin{array}{c} \alpha_{1}\\ \alpha_{2} \end{array}\right),$$

where *α*_3_ = 0 has been substituted (i.e. no nonlinear spring grounding the second mass) and where *ϕ*_ij_ are elements of the linear modeshape matrix ***Φ***, defined as

3.2 $$\Phi =\left[\begin{array}{cc} \phi_{11} & \phi_{12}\\ \phi_{21} & \phi_{22} \end{array}\right].$$

Note that the first column of this matrix, i.e. *ϕ*_11_ and *ϕ*_21_, represents the modeshape of the first linear mode, while the second linear modeshape is captured by *ϕ*_12_ and *ϕ*_22_, in the second column of ***Φ***. In order for *Ψ*_1_ = 0 and *Ψ*_2_ = 0 to be satisfied, equation (3.1) shows that either *α*_1_ = 0 and *α*_2_ = 0 (which would represent the trivial case where the system is linear), or that the determinant of the matrix in equation (3.1) must be zero, i.e.

3.3 $$\phi_{11}^{3}\phi_{12}\left(\phi_{11}-\phi_{21}\right){\left(\phi_{12}-\phi_{22}\right)}^{3}-\phi_{11}\phi_{12}^{3}{\left(\phi_{11}-\phi_{21}\right)}^{3}\left(\phi_{12}-\phi_{22}\right)=0.$$

Note that for a system with an asymmetric configuration, *ϕ*_ij_ are non-zero, and *ϕ*_11_≠*ϕ*_21_ and *ϕ*_12_≠*ϕ*_22_. Thus, equation (3.3) can be rearranged to

3.4 $$\frac{\phi_{11}^{2}{\left(\phi_{12}-\phi_{22}\right)}^{2}}{\phi_{12}^{2}{\left(\phi_{11}-\phi_{21}\right)}^{2}}=1,$$

which can be satisfied using the following conditions:

3.5*a* $$\frac{\phi_{11}\left(\phi_{12}-\phi_{22}\right)}{\phi_{12}\left(\phi_{11}-\phi_{21}\right)}=1:\;\frac{\phi_{11}}{\phi_{21}}-\frac{\phi_{12}}{\phi_{22}}=0$$

and

3.5*b* $$\frac{\phi_{11}\left(\phi_{12}-\phi_{22}\right)}{\phi_{12}\left(\phi_{11}-\phi_{21}\right)}=-1:\;\frac{\phi_{21}}{\phi_{11}}+\frac{\phi_{22}}{\phi_{12}}=2.$$

Condition (3.5a) cannot be satisfied as it requires that the first and second modeshapes are equal; therefore, dynamic symmetry, i.e. when *Ψ*_1_ = *Ψ*_2_ = 0, may only be achieved when condition (3.5b) is satisfied. The relationship between the modeshape coefficients, *ϕ*_ij_, and the linear physical parameters is derived in appendix A. Substituting equations (A 7) into condition (3.5b) (also using equation (A 6)) leads to

3.6 $$\frac{k_{1}}{k_{2}}=\frac{m_{1}+m_{2}}{m_{2}}.$$

This demonstrates that, in order for the NLTMD-inspired system to exhibit dynamic symmetry, the linear stiffness coefficients must obey the ratio described by equation (3.6). As well as this condition for the linear parameters, the relationship between the nonlinear parameters may be found by substituting expressions (3.6) and (A 7) back into equation (3.1), leading to

3.7 $$\frac{\alpha_{1}}{\alpha_{2}}=\frac{{\left(\phi_{12}-\phi_{22}\right)}^{4}}{\phi_{12}^{4}}=\frac{{\left(\phi_{11}-\phi_{21}\right)}^{4}}{\phi_{11}^{4}}={\left(\frac{m_{1}+m_{2}}{m_{2}}\right)}^{2}.$$

**Figure 6.**
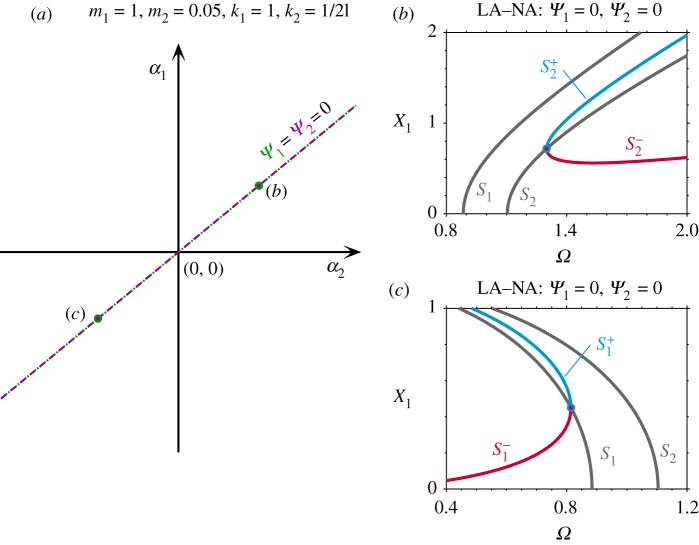
Dynamic symmetry for an NLTMD-inspired system. (*a*) The nonlinear parameter space, *α*_1_ against *α*_2_, for a system with LA, i.e. m_1_ = 1, m_2_ = 0.05, k_1_ = 1 and k_2_ = 1/21, where *Ψ*_1_ = 0 and *Ψ*_2_ = 0 are two overlapping lines. (*b*) Backbone curves with dynamic symmetry for a hardening LA–NA system when *α*_1_ = 1 and *α*_2_ = 1/441 (represented by a solid dot labelled (*b*) in panel (*a*)). (*c*) Backbone curves with dynamic symmetry for a softening LA–NA system when *α*_1_ = − 1 and *α*_2_ = − 1/441 (represented by a solid dot labelled (*c*) in panel (*a*)). (Online version in colour.)

Figure 6*a* shows the nonlinear parameter space *α*_1_ against *α*_2_, for the case where k_1_/k_2_ = 21, and where the mass values satisfy equation (3.6). The overlapping green and purple lines represent the parameter relationships that lead to *Ψ*_1_ = 0 and *Ψ*_2_ = 0, respectively (in this instance the case *α*_1_/*α*_2_=441, satisfies equation (3.7) and hence *Ψ*_1_ = *Ψ*_2_ = 0). Despite being a linearly asymmetric system, this has strong similarities to figure 2*a*, which represents an LS system, and indicates that multiple nonlinear parameter combinations will lead to dynamic symmetry. Note that when the parameter relationships (3.6) and (3.7) are satisfied, expressions for *Ψ*_1_, …, *Ψ*_5_ (A 5) can be simplified as

3.8 $$\Psi_{1}=0,\;\Psi_{2}=0,\;\Psi_{3}=6\phi_{11}^{2}\phi_{12}^{2}\alpha_{1},\;\Psi_{4}=2\phi_{11}^{4}\alpha_{1},\;\Psi_{5}=2\phi_{12}^{4}\alpha_{1}.$$

With *Ψ*_1_ = 0 and *Ψ*_2_ = 0, expressions for backbone curves (2.6) can be reduced to

3.9*a* $$\begin{array}{rl} & \left\{4\left(\omega_{n1}^{2}-\Omega^{2}\right)+3\Psi_{4}U_{1}^{2}+\Psi_{3}U_{2}^{2}\left[1+2\cos^{2}\left(\theta_{d}\right)\right]\right\}U_{1}=0, \end{array}$$

3.9*b* $$\begin{array}{rl} & \left\{4\left(\omega_{n2}^{2}-\Omega^{2}\right)+3\Psi_{5}U_{2}^{2}+\Psi_{3}U_{1}^{2}\left[1+2\cos^{2}\left(\theta_{d}\right)\right]\right\}U_{2}=0 \end{array}$$

3.9*c* $$\begin{array}{rl} \;\mathrm{and}\; & 2\Psi_{3}U_{1}U_{2}\cos\left(\theta_{d}\right)\sin\left(\theta_{d}\right)=0. \end{array}$$

The case where U_1_ = 0 and U_2_ = 0 represent the trivial case where the system is stationary. Two sets of single-mode solutions, denoted S_1_ and S_2_, can be found with frequency–amplitude relationships described as

3.10 $$S_{1}:\;U_{2}=0,\;\Omega^{2}=\omega_{n1}^{2}+\frac{3}{4}\Psi_{4}U_{1}^{2}$$

and

3.11 $$S_{2}:\;U_{1}=0,\;\Omega^{2}=\omega_{n2}^{2}+\frac{3}{4}\Psi_{5}U_{2}^{2}.$$

This system can also exhibit mixed-mode backbone curves. To compute these, the phase relationship, *θ*_d_, between the fundamental components of the two modal coordinates, u_1_ and u_2_, needs to be determined. From equation (3.9c), this may be satisfied when *θ*_d_ = n*π*/2, with n∈Z. The case where n is odd, which satisfies cos(*θ*_d_) = 0, represents solutions exhibiting out-of-unison resonance [46], i.e. the two modes are ±90° out-of-phase. The case where n is even, satisfying sin(*θ*_d_) = 0, represents in-phase and anti-phase solutions. Considering the out-of-unison solutions, the substitution of cos(*θ*_d_) = 0 into equations (3.9a) and (3.9b), leads to the frequency–amplitude relationship

3.12*a* $$S_{1,2}^{\pm 90}:\;U_{1}^{2}=\frac{4\left(\omega_{n2}^{2}-\omega_{n1}^{2}\right)+\left(3\Psi_{5}-\Psi_{3}\right)U_{2}^{2}}{3\Psi_{4}-\Psi_{3}}$$

and

3.12*b* $$\Omega^{2}=\frac{4\left(3\Psi_{4}\omega_{n2}^{2}-\Psi_{3}\omega_{n1}^{2}\right)+\left(9\Psi_{4}\Psi_{5}-\Psi_{3}^{2}\right)U_{2}^{2}}{4\left(3\Psi_{4}-\Psi_{3}\right)}.$$

With substitution of expressions (3.6), (3.8), (A 6) and (A 8) into equation (3.12b), one can find that the response frequency, *Ω*, of out-of-unison resonance, in this case, is always zero. This means that out-of-unison solutions cannot exist in the dynamically symmetric case for this system.

The in-phase solutions, corresponding to *θ*_d_ = 0 are denoted S^+^_1_ and S^+^_2_, while the anti-phase solutions, corresponding to *θ*_d_ = *π* are denoted S^−^_1_ and S^−^_2_ (S^±^_1,2_ is used to denote all of them). For the dynamically symmetric case, these backbone curves all share the frequency-amplitude relationship, given by

3.13*a* $$S_{1,2}^{\pm }:\;U_{1}^{2}=\frac{4\left(\omega_{n2}^{2}-\omega_{n1}^{2}\right)+3\left(\Psi_{5}-\Psi_{3}\right)U_{2}^{2}}{3\left(\Psi_{4}-\Psi_{3}\right)}$$

and

3.13*b* $$\Omega^{2}=\frac{4\left(\Psi_{4}\omega_{n2}^{2}-\Psi_{3}\omega_{n1}^{2}\right)+3\left(\Psi_{4}\Psi_{5}-\Psi_{3}^{2}\right)U_{2}^{2}}{4\left(\Psi_{4}-\Psi_{3}\right)}.$$

As well as two single-mode backbone curves, described by equations (3.10) and (3.11), dynamic symmetry also requires two mixed-mode backbone curves, described by equations (3.13), with a perfect bifurcation on either S_2_, which are denoted S^±^_2_, or on S_1_, which are denoted S^±^_1_. For the perfect bifurcation point on S_2_, the amplitude of the first modal coordinate U_1_ = 0; likewise, the second modal amplitude U_2_ = 0 for the perfect bifurcation point on S_1_. Using these conditions, the amplitude and frequency of these two bifurcation points can be obtained, from equations (3.13), as

3.14*a* $$\text{bifurcation point on}\;S_{1}:\;U_{1}^{2}=\frac{4\left(\omega_{n2}^{2}-\omega_{n1}^{2}\right)}{3\left(\Psi_{4}-\Psi_{3}\right)},\;\Omega^{2}=\frac{\Psi_{4}\omega_{n2}^{2}-\Psi_{3}\omega_{n1}^{2}}{\Psi_{4}-\Psi_{3}}$$

and

3.14*b* $$\text{bifurcation point on}\;S_{2}:\;U_{2}^{2}=\frac{4\left(\omega_{n2}^{2}-\omega_{n1}^{2}\right)}{3\left(\Psi_{3}-\Psi_{5}\right)},\;\Omega^{2}=\frac{\Psi_{3}\omega_{n2}^{2}-\Psi_{5}\omega_{n1}^{2}}{\Psi_{3}-\Psi_{5}}.$$

As *ω*_n2_ > *ω*_n1_, to obtain positive solutions, i.e. positive amplitude and frequency, requires

3.15*a* $$\text{bifurcation point on}\;S_{1}:\;\Psi_{4}-\Psi_{3}>0,\;\Psi_{4}\omega_{n2}^{2}-\Psi_{3}\omega_{n1}^{2}>0$$

and

3.15*b* $$\text{bifurcation point on}S_{2}:\;\Psi_{3}-\Psi_{5}>0,\;\Psi_{3}\omega_{n2}^{2}-\Psi_{5}\omega_{n1}^{2}>0.$$

Note that conditions (3.15) are valid for any system with cubic nonlinearities and a 1 : 1 resonance between two modes. To relate these to the NLTMD system, the expressions for modeshape elements (A 8) and the nonlinear parameter relationship (3.7) are substituted into these inequalities. This reveals that a perfect bifurcation from S_1_ onto S^±^_1_ exists when both nonlinear parameters are softening, i.e. *α*_1_ < 0 and *α*_2_ < 0; while a perfect bifurcation from S_2_ onto S^±^_2_ may be seen for hardening nonlinear parameters, i.e. *α*_1_ > 0 and *α*_2_ > 0 when m_2_ < m_1_/3. Figure 6*b*,*c* shows the backbone curves with dynamic symmetry, i.e. satisfying parameter conditions (3.6), (3.7) and (3.15) for systems with hardening and softening parameters, respectively (labelled with (*b*) and (*c*), respectively, in figure 6*a*).

### (b) Evolution of backbone curves in the nonlinear parameter space

In the previous discussion, we did not consider the tuning of the NLTMD, but rather concentrated on whether a solution exists that exhibits dynamic symmetry and derived the linear and nonlinear parameter relationships, (3.6), (3.7) and (3.15) for this to occur. Now, to investigate the evolution of backbone curves in nonlinear parameter space, it is assumed that the linear parameters of the NLTMD are tuned to achieve optimal performance.

The classical approach for optimizing the linear parameters of a TMD is known as the fixed-points method [18]. Instead of imposing two fixed points, using H_∞_ optimization, a closed-form exact solution to obtain equal peaks in receptance curves of the underlying linear system is discussed in [47], where the linear stiffness of the NLTMD can be optimized using

3.16 $$k_{2}^{\mathrm{opt}}=\frac{8\mu k_{1}\left[16+23\mu +9\mu^{2}+2\left(2+\mu \right)\sqrt{4+3\mu }\right]}{3{\left(1+\mu \right)}^{2}\left(64+80\mu +27\mu^{2}\right)},$$

where *μ* = m_2_/m_1_ is the mass ratio and k^opt^_2_ is the optimized linear spring coefficient of the NLTMD. This cannot satisfy relationship (3.6), and hence dynamic symmetry cannot be achieved. However, if nonlinear parameters are correspondingly selected on either *Ψ*_1_ = 0 or *Ψ*_2_ = 0, single-mode backbone curves, S_1_ and S_2_, respectively, can still be solved via amplitude–frequency relationships (3.10) and (3.11). To find the nonlinear parameter conditions that lead to either *Ψ*_1_ = 0 or *Ψ*_2_ = 0, the modeshape expressions (A 7) are substituted into the expressions for *Ψ*_i_ (A 5) (with *α*_3_ = 0). Letting *Ψ*_1_ = 0 and *Ψ*_2_ = 0, respectively, one has

3.17*a* $$\Psi_{1}=0:\;\frac{\alpha_{1}}{\alpha_{2}}=-\frac{{\left(-\omega_{n1}^{2}m_{1}+k_{1}\right)}^{3}\left(-\omega_{n2}^{2}m_{1}+k_{1}\right)}{k_{2}^{4}}$$

and

3.17*b* $$\Psi_{2}=0:\;\frac{\alpha_{1}}{\alpha_{2}}=-\frac{\left(-\omega_{n1}^{2}m_{1}+k_{1}\right){\left(-\omega_{n2}^{2}m_{1}+k_{1}\right)}^{3}}{k_{2}^{4}}.$$

**Figure 7.**
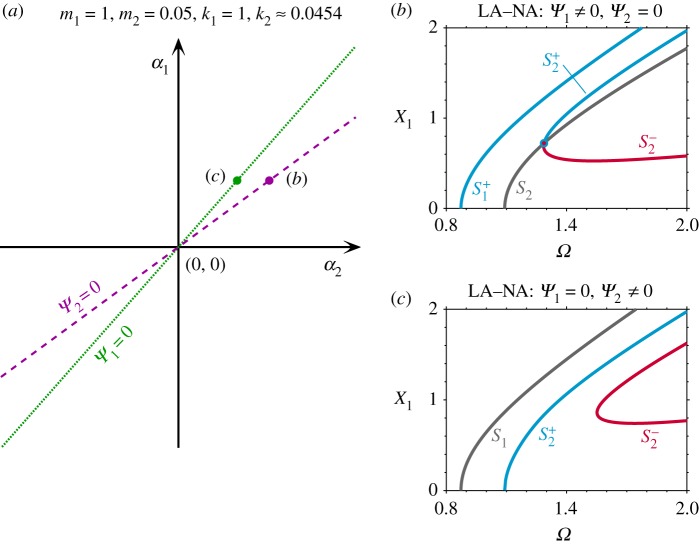
Optimizing the linear parameters of the NLTMD leads to the breaking of dynamic symmetry for an NLTMD-inspired system. (*a*) Nonlinear parameter space, *α*_1_ against *α*_2_, for a system with LA, i.e. m_1_ = 1, m_2_ = 0.05, k_1_ = 1 and k_2_ = k^opt^_2_ ≈ 0.0454. The *α*_1_ and *α*_3_ values that lead to *Ψ*_1_ = 0 and *Ψ*_2_ = 0 are shown as a green dotted line and a purple dashed line, respectively, and curves *Ψ*_1_ = 0 and *Ψ*_2_ = 0 are intersecting at the origin. (*b*) Backbone curves with the single-mode solution S_2_ for the LA–NA system when *α*_1_ = 1 and *α*_2_ ≈ 0.00256 (represented by a purple dot labelled (*b*) in panel (*a*)). (*c*) Backbone curves with the single-mode backbone curve S_1_ for the LA–NA system when *α*_1_ = 1 and *α*_2_ ≈ 0.00166 (represented by a green dot labelled (*c*) in panel (*a*)). (Online version in colour.)

As depicted in figure 7*a*, the curves *Ψ*_1_ = 0 and *Ψ*_2_ = 0 do not overlap, instead, these curves now are intersecting at the origin in the nonlinear parameter space (where the system is reduced to a linear one). Backbone curves for the system with *Ψ*_1_ = 0 and *Ψ*_2_≠0 (the point labelled (*c*) in figure 7*a*) are shown in figure 7*c*; these are similar to those shown in figure 4*c*, where a single-mode backbone curve S_1_ is also present. Likewise, backbone curves for the system with *Ψ*_2_ = 0 and *Ψ*_1_≠0 (the point labelled (*b*) in figure 7*a*) are shown in figure 7*b*, which have similarity to figure 4*b*.

Systems with nonlinear parameters that do not lie on *Ψ*_1_ = 0 or *Ψ*_2_ = 0 exhibit mixed-mode backbone curves. As previously, the phase relationship between the two modal coordinates needs to be determined using equation (2.6c). This expression is satisfied with real and positive solutions by sin(*θ*_d_) = 0, leading to the phase relationship *θ*_d_ = *θ*_1_ − *θ*_2_ = n*π*, where even and odd n values denote in-phase and anti-phase modal relationships, respectively. Further defining the phase parameter p as

3.18 $$p=\cos\left(\theta_{d}\right)=\cos\left(n\pi \right)=\left\{ \begin{array}{ll} +1 & \text{for even }\;n\\ -1 & \text{for odd }\;n, \end{array}\right.$$

allows the expressions governing the modal amplitudes and frequencies, given in (2.6a,b), to be written

3.19*a* $$4\left(\omega_{n1}^{2}-\Omega^{2}\right)U_{1}+3\left[\Psi_{4}U_{1}^{3}+\Psi_{3}U_{1}U_{2}^{2}+p\left(\Psi_{2}U_{2}^{3}+3\Psi_{1}U_{1}^{2}U_{2}\right)\right]=0$$

and

3.19*b* $$4\left(\omega_{n2}^{2}-\Omega^{2}\right)U_{2}+3\left[\Psi_{5}U_{2}^{3}+\Psi_{3}U_{1}^{2}U_{2}+p\left(\Psi_{1}U_{1}^{3}+3\Psi_{2}U_{1}U_{2}^{2}\right)\right]=0.$$

Rearranging these two equations gives the frequency-amplitude relationships

3.20*a* $$\Omega^{2}=\omega_{n1}^{2}+\frac{3}{4}\left[\Psi_{4}U_{1}^{3}+\Psi_{3}U_{2}^{2}U_{1}+p\left(\Psi_{2}U_{2}^{3}+3\Psi_{1}U_{1}^{2}U_{2}\right)\right]U_{1}^{-1}$$

and

3.20*b* $$\begin{array}{rl} 0 & =\left(-3p\Psi_{2}U_{1}^{-1}\right)U_{2}^{4}+3\left(\Psi_{5}-\Psi_{3}\right)U_{2}^{3}+\left[9p\left(\Psi_{2}-\Psi_{1}\right)U_{1}\right]U_{2}^{2}\\ & \;+\left[4\omega_{n2}^{2}-4\omega_{n1}^{2}+3\left(\Psi_{3}-\Psi_{4}\right)U_{1}^{2}\right]U_{2}+3p\Psi_{1}U_{1}^{3}, \end{array}$$

which may be solved to find the mixed-mode backbone curves.

**Figure 8.**
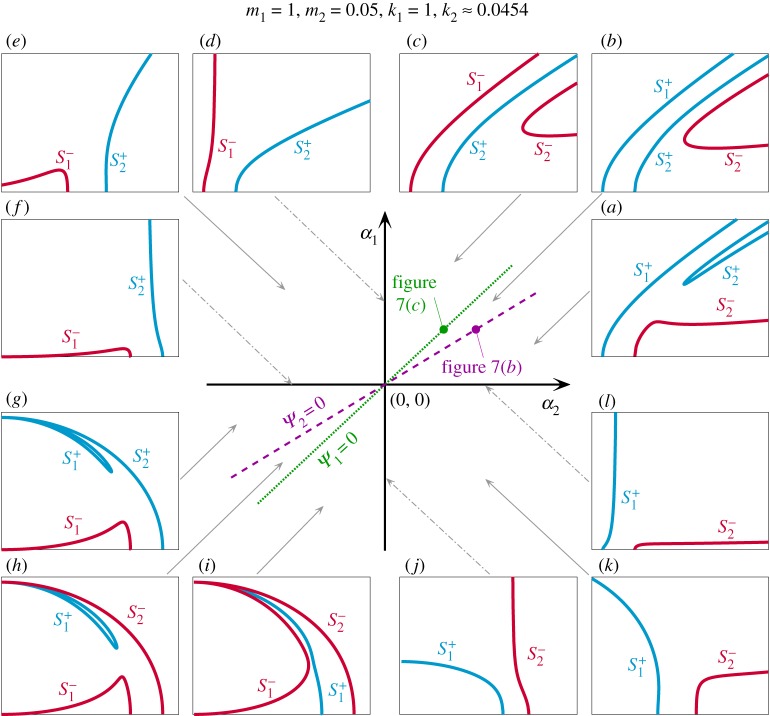
The evolution of backbone curves in the nonlinear parameter space, *α*_1_ against *α*_2_, for a system with LA, i.e. m_1_ = 1, m_2_ = 0.05, k_1_ = 1 and k_2_ = k^opt^_2_ ≈ 0.0454. The *α*_1_ and *α*_2_ values that lead to *Ψ*_1_ = 0 and *Ψ*_2_ = 0 are shown as a green dotted line and a purple dashed line, respectively. The panels around the main figure show backbone curves topologies for the nonlinear regions in terms of response frequency and displacement amplitude of the first mass. Panels (*a*,*b*,*c*,*e*,*g*,*h*,*i*,*k*) show these topologies in regions indicated by the solid-grey arrows. Panels (*d*,*f* ,*j*,*l*) show the topologies corresponding to *α*_1_ and *α*_2_ axes, as indicated by the dash-dotted grey arrows. (Online version in colour.)
  Further varying the nonlinear parameters in the anticlockwise direction towards *Ψ*_1_ = 0, the contribution of the second modal coordinate, U_2_, to the mixed-mode, in-phase backbone curve, S^+^_1_, gradually decreases to zero. This results in a single-mode backbone curve S_1_, seen from the evolution of backbone curves from figure 8*b* to 7*c*. Finally, perturbing anticlockwise from *Ψ*_1_ = 0, the U_2_ component of S_1_ increases, leading to a mixed-mode, anti-phase backbone curve S^−^_1_, as shown in figure 8*c*.
(ii) **a hardening structure with a softening attachment (the second quadrant)**
  Further decreasing *α*_2_ until *α*_2_ < 0, causes S^−^_1_ to bend leftward, as depicted in figure 8*e*. Note that no isolated backbone curve is predicted for systems in the second quadrant.
(iii) **a softening system (the third quadrant)**
  Crossing from the second quadrant into the third causes S^+^_2_ to bend leftward, along with S^−^_1_. Continuing anticlockwise, from above *Ψ*_2_ = 0 to below it, leads to a similar behaviour to the hardening system (the first quadrant) as it crosses *Ψ*_1_ = 0. The contribution from U_1_ to the mixed-mode, in-phase backbone curve S^+^_2_ may gradually decrease, reaching zero at *Ψ*_2_ = 0, leading to a single-mode backbone curve S_2_. The contribution then increases, resulting in a mixed-mode, anti-phase backbone curve S^−^_2_. Such behaviour is shown in figure 8*g*,*h*. Simultaneously, the isolated backbone curve, S^+^_1_, emerges from zero frequency and draws closer to the primary backbone curve S^−^_1_.
  Further varying the nonlinear parameters towards *Ψ*_1_ = 0, the isolated and primary backbone curves, S^+^_1_ and S^−^_1_, merge into a single-mode backbone curve, S_1_, with a perfect bifurcation. Anticlockwise of *Ψ*_1_ = 0, the perfect bifurcation breaks and generates an isolated backbone curve, S^−^_1_, below two primary backbone curves, S^+^_1_ and S^−^_2_, shown in figure 8*i*.
(iv) **a softening structure with a hardening attachment (the fourth quadrant)**
  Crossing from the third to the fourth quadrant leads to the disappearance of the isolated backbone curve, S^−^_1_, at zero frequency, as shown in figure 8*i*,*j*. Additionally, the mixed-mode backbone curve, S^−^_2_, bends rightward, shown in figure 8*j*,*k*.

Figure 8 presents the mixed-mode backbone curves of systems with linear parameters m_1_ = 1, m_2_ = 0.05, k_1_ = 1, k_2_ = k^opt^_2_ ≈ 0.0454 in the nonlinear parameter space *α*_1_ against *α*_2_. Backbone curves on *Ψ*_1_ = 0 and *Ψ*_2_ = 0, labelled in this figure, are the same as those in figures 7*c*,*b*, respectively. This space is divided by the parameter axes *α*_1_ = 0 and *α*_2_ = 0 into the following four classes of system:

(i) **a hardening system (the first quadrant)**
  Perturbing the nonlinear parameters clockwise from *Ψ*_2_ = 0 breaks the perfect bifurcation on S_2_, shown in figure 7*b*. The breaking of this bifurcation generates one isolated backbone curve, S^+^_2_, between two primary backbone curves, S^−^_2_ and S^+^_1_, ^4^ seen in figure 8*a*. Likewise, if nonlinear parameters are perturbed anticlockwise from *Ψ*_2_ = 0, the perfect bifurcation on S_2_ breaks in a different direction, resulting in one isolated backbone curve, S^−^_2_, below two primary backbone curves, depicted in figure 8*b*.

For further demonstration of the evolution of backbone curves in nonlinear parameter space, see the video, *Evolution of backbone curves.avi*, provided as electronic supplementary material.

From figure 8, it can be seen that the hardening systems (the first quadrant) and softening systems (the third quadrant) share the following features:

(i) **a change of contribution of, and phase relationship between, two modal coordinates**, from being in-phase, to single-mode, and then to anti-phase, or vice versa, when crossing *Ψ*_1_ = 0 and *Ψ*_2_ = 0;
(i1) **the emergence and breaking of a perfect bifurcation** on S_2_ for a hardening system when crossing *Ψ*_2_ = 0, and on S_1_ for a softening system when crossing *Ψ*_1_ = 0.

The perfect bifurcations denote critical conditions, perturbing from which leads to the onset of isolated backbone curves. These conditions are defined by relationships (3.17a,b) and thus represent the boundaries for the existence of isolated backbone curves. The following section explores additional boundaries that may exist.

## 4. Additional topological boundaries

**Figure 9.**
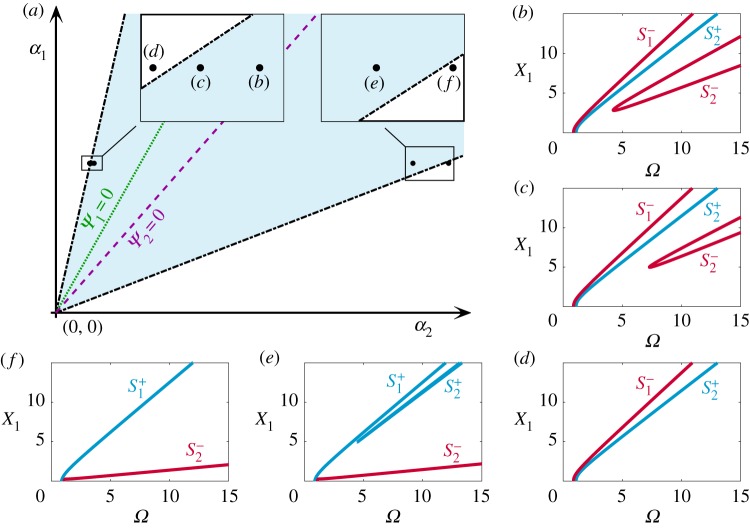
Two additional topological boundaries for the existence of isolated backbone curves. (*a*) The first quadrant of figure 8, along with two additional boundaries, shown as dash-dotted black lines, differentiating between regions with and without isolated backbone curves. Panels (*b*), …, (*f* ) are backbone curves of systems represented by black dots in Panel (a) for a system with *α*_1_ = 1 and varied *α*_2_. (Online version in colour.)

As described in §3b, a perturbation from *Ψ*_2_ = 0 breaks the perfect bifurcation on S_2_, for a hardening system, and results in an isolated backbone curve, shown in figures 7*b* and 8*a*,*b*. Further deviation may cause the isolated backbone curve to move toward higher frequency and larger amplitude, as depicted in figure 9*b*,*c*, which are corresponding to systems on points labelled (*b*) and (*c*), respectively in figure 9(*a*); eventually, the isolated backbone curve may undergo swift change from finite frequency and amplitude to infinite values. One example of this change is depicted in figure 9*b*–*d*. The isolated backbone curve first increases in frequency and amplitude at a limited rate, seen from figure 9*b*,*c* as *α*_2_ changes from 7.5 × 10^−4^ to 7.0 × 10^−4^. It then shifts to infinite frequency and amplitude as *α*_2_ approaches a critical value of approximately 6.76 × 10^−4^, shown in figure 9*d*. This corresponds to the vanishing (or emergence, if *α*_2_ is increased) of an isolated backbone curve, and this critical value defines another topological difference in backbone curves, i.e. systems with and without an isolated backbone curve.

To find the conditions defining such boundaries in nonlinear parameter space, one can trace the isolated backbone curve to seek conditions for its existence. It is observed that the isolated backbone curve vanishes when the amplitude of the minimum frequency solution becomes infinite; hence, the conditions that lead to this case are investigated here.^5^ Since the minimum frequency solution is related to a multiple root of amplitude for the frequency–amplitude equations (3.20), one can refer to the zero discriminant of the amplitude equation (3.20b) to trace the multiple root. The zero discriminant of the quartic equation (3.20b) is a sixth-order polynomial equation with respect to U_1_, and it can be written as

4.1 $${\mathrm{Disc}}_{U_{2}}=0:\;f_{6}U_{1}^{6}+f_{5}U_{1}^{5}+f_{4}U_{1}^{4}+f_{3}U_{1}^{3}+f_{2}U_{1}^{2}+f_{1}U_{1}+f_{0}=0,$$

where f_6_ is a function of nonlinear parameters, written as

4.2 $$f_{6}=g_{1}\alpha_{1}^{6}+g_{2}\alpha_{1}^{5}\alpha_{2}+g_{3}\alpha_{1}^{4}\alpha_{2}^{2}+g_{4}\alpha_{1}^{3}\alpha_{2}^{3}+g_{5}\alpha_{1}^{2}\alpha_{2}^{4}+g_{6}\alpha_{1}\alpha_{2}^{5}+g_{7}\alpha_{2}^{6},$$

and where coefficients g_1_, …, g_7_ are determined by the underlying linear system, some of which are given in appendix B. Note that f_0_, …, f_5_, g_1_, g_2_, g_6_ and g_7_ are not provided as they are not required for the following derivations.

As the isolated backbone curve reaches the vanishing point, it has infinite amplitude; thus, letting $U_{1}\to \infty$, gives

4.3 $${\mathrm{Disc}}_{U_{2}}\approx f_{6}=0.$$

After some algebraic manipulation, one can find coefficients g_1_ and g_7_ have factors (p − 1)^2^(p + 1)^2^, while coefficients g_2_ and g_6_ have factors (p − 1)(p + 1). Recalling that p = ± 1, defined in expression (3.18), it follows that g_1_ = g_7_ = g_2_ = g_6_ = 0, and equation (4.3) can be further simplified to give

4.4 $$f_{6}=\alpha_{1}^{2}\alpha_{2}^{2}\left(g_{3}\alpha_{1}^{2}+g_{4}\alpha_{1}\alpha_{2}+g_{5}\alpha_{2}^{2}\right)=0.$$

Two non-zero solutions for *α*_2_ are

4.5 $$\alpha_{2}=\frac{-g_{4}\pm \sqrt{g_{4}^{2}-4g_{5}g_{3}}}{2g_{5}}\alpha_{1}.$$

Likewise, if frequency–amplitude relationships (3.20) are rearranged to give a quartic amplitude equation with respect to U_1_ rather than in U_2_, as currently, one can find same expression as (4.5) by following the procedure demonstrated above. This means U_1_ and U_2_ will shift to infinity simultaneously on the critical conditions described by expression (4.5). As *Ω* is explicitly determined by equation (3.20a), it will also shift to infinity when $U_{1}\to \infty$ and $U_{2}\to \infty$.

Equation (4.5) represents conditions between *α*_1_ and *α*_2_ when the isolated backbone curve has infinite frequency and amplitude. This allows the first quadrant in figure 8, i.e. the hardening system, to be further divided into additional regions, as shown in figure 9*a*. The new regions anticlockwise of *Ψ*_1_ = 0 describe:

(i) the shaded area anticlockwise of *Ψ*_1_ = 0: two primary backbone curves, S^−^_1_ and S^+^_2_, with one isolated backbone curve, S^−^_2_, below those two—shown in figure 9*b*,*c*;
(ii) the unshaded area anticlockwise of *Ψ*_1_ = 0: two primary backbone curves, S^−^_1_ and S^+^_2_, without an isolated backbone curve—depicted in figure 9*d*.

The new regions clockwise of *Ψ*_2_ = 0 describe:

(i) the shaded area clockwise of *Ψ*_2_ = 0: two primary backbone curves, S^+^_1_ and S^−^_2_, with one isolated backbone curve, S^+^_2_, between those two—shown in figure 9*e*;
(ii) the unshaded area clockwise of *Ψ*_2_ = 0: two primary backbone curves, S^+^_1_ and S^−^_2_, without an isolated backbone curve—depicted in figure 9*f* .

In summary, expressions (3.17a,b), combined with conditions (3.15), are boundaries for the existence of a perfect bifurcation for a hardening and a softening system, respectively, perturbing from which the bifurcation breaks and an isolated backbone curve emerges. Expressions (4.5) describe the other boundaries at which isolated backbone curves may vanish or emerge from infinite frequency and amplitude. The shaded area in figure 9*a* highlights the region in which an isolated backbone curve can exist.

## 5. Conclusion

Isolated backbone curves can be related to isolated forced responses, which can have a significant negative impact on the performance of nonlinear engineering systems. This paper has investigated the conditions for the existence of isolated backbone curves of a two-mode system with cubic nonlinearities and a 1 : 1 resonance. The concept of *dynamic symmetry* has been defined as the case where a system exhibits two single-mode backbone curves with one perfect bifurcation. By breaking the symmetry of a simple example system, we have found that dynamic symmetry is still obtainable when the system is asymmetric. This highlights that an asymmetric system may exhibit dynamic behaviour that is equivalent to that of a symmetric system. A specific two-mode asymmetric system, composed of a primary structure and an NLTMD, was then considered, and an analytical approach was used to demonstrate that dynamic symmetry may only be achieved when the linear parameters obey specific relationships. After optimizing the linear parameters for vibration suppression performance, we have demonstrated analytical methods that allow the nonlinear parameter space to be divided into several regions, within which backbone curves present similar topological features. The boundaries of these regions define conditions for the existence of the isolated backbone curves. We have then demonstrated how these regions can be further refined by considering whether the isolated backbone curves can exist for finite amplitudes and frequencies.

The methodology used in this paper is based on a general two-mode model with cubic nonlinearities and a 1 : 1 internal resonance. While specific example systems have been considered, the approach used may be generalized to similar systems. This allows the existence of isolated backbone curves to be determined more rigorously when designing nonlinear systems.

## Supplementary Material

Video showing the evolution of backbone curves with parameter space

## Footnotes

1 Note the distinction between isolated backbone curves and *isolas*, which exist in the forced responses.

2 Note that the term *mode* is used here to refer to a mode of the underlying linear model of the system, whereas a *Nonlinear Normal Mode* (NNM) denotes a periodic response of the conservative nonlinear system [5].

3 Other analytical methods, such as the second-order normal form technique [44] or the multiple-scales method [45], could alternatively be used.

4 Note that in other projections, such as *Ω* against X_2_, the relative amplitudes of these backbone curves may differ.

5 There may be other conditions that allow isolated backbone curves to vanish/emerge; however, this particular case is investigated here as an example of such behaviour, rather than as an exhaustive study.

## Data accessibility

This article has no additional data.

## Appendix A. Modal analysis for the two-mass oscillator in §2

The equation of motion of the system shown in figure 1 is written as

A 1 $$M\ddot{x}+Kx+N_{x}=0,$$

where **M** and **K** are mass and linear stiffness matrices, respectively, **N**_**x**_ is a vector of nonlinear stiffness terms, and **x** is a vector representing physical displacements. They are written

A 2 $$\begin{array}{rl} & M=\left[\begin{array}{cc} m_{1} & 0\\ 0 & m_{2} \end{array}\right],\;K=\left[\begin{array}{cc} k_{1}+k_{2} & -k_{2}\\ -k_{2} & k_{2}+k_{3} \end{array}\right]\\ \;\mathrm{and}\; & N_{x}=\left(\begin{array}{c} \alpha_{1}x_{1}^{3}+\alpha_{2}{\left(x_{1}-x_{2}\right)}^{3}\\ \alpha_{2}{\left(x_{2}-x_{1}\right)}^{3}+\alpha_{3}x_{2}^{3} \end{array}\right),\;x=\left(\begin{array}{c} x_{1}\\ x_{2} \end{array}\right). \end{array}\}$$

The system is translated into linear modal space by substituting **x** = ***Φ*****q** into equations (A 1), where ***Φ*** is the linear modeshape matrix, which is written

A 3 $$\Phi =\left[\begin{array}{cc} \phi_{11} & \phi_{12}\\ \phi_{21} & \phi_{22} \end{array}\right].$$

Further multiplying both sides by ***Φ***^T^, the equations of motion can be obtained as

A 4 $$\Phi^{T}M\Phi \ddot{q}+\Phi^{T}K\Phi q+N_{q}=0$$

where **N**_**q**_ = ***Φ***^T^**N**_**x**_(***Φ*****q**) and ***Φ***^T^**M*****Φ*** = **I**. As can be seen, after the linear modal transform, one can obtain the equations of motion in the same form as equations (2b) with

A 5 $$\begin{array}{rl} & \Psi_{1}=\phi_{11}^{3}\phi_{12}\alpha_{1}+{\left(\phi_{11}-\phi_{21}\right)}^{3}\left(\phi_{12}-\phi_{22}\right)\alpha_{2}+\phi_{21}^{3}\phi_{22}\alpha_{3},\\ & \Psi_{2}=\phi_{11}\phi_{12}^{3}\alpha_{1}+\left(\phi_{11}-\phi_{21}\right){\left(\phi_{12}-\phi_{22}\right)}^{3}\alpha_{2}+\phi_{21}\phi_{22}^{3}\alpha_{3},\\ & \Psi_{3}=3\left[\phi_{11}^{2}\phi_{12}^{2}\alpha_{1}+{\left(\phi_{11}-\phi_{21}\right)}^{2}{\left(\phi_{12}-\phi_{22}\right)}^{2}\alpha_{2}+\phi_{21}^{2}\phi_{22}^{2}\alpha_{3}\right],\\ & \Psi_{4}=\phi_{11}^{4}\alpha_{1}+{\left(\phi_{11}-\phi_{21}\right)}^{4}\alpha_{2}+\phi_{21}^{4}\alpha_{3}\\ \;\mathrm{and}\; & \Psi_{5}=\phi_{12}^{4}\alpha_{1}+{\left(\phi_{12}-\phi_{22}\right)}^{4}\alpha_{2}+\phi_{22}^{4}\alpha_{3}. \end{array}\}$$

To interpret the modeshape elements *ϕ*_ij_ by physical parameters, the linear modal analysis is then carried out by finding the eigenvalues and eigenvectors of **M**^−1^**K**, leading to

A 6 $$\omega_{ni}^{2}=\frac{\left[(k_{2}+k_{3})/m_{2}+(k_{1}+k_{2})/m_{1}\right]\pm \sqrt{{\left[(k_{2}+k_{3})/m_{2}-(k_{1}+k_{2})/m_{1}\right]}^{2}+4(k_{2}/m_{1})(k_{2}/m_{2})}}{2},$$

and

A 7*a* $$\phi_{11}^{2}=\frac{k_{2}^{2}}{{\left(k_{1}+k_{2}-m_{1}\omega_{n1}^{2}\right)}^{2}m_{2}+m_{1}k_{2}^{2}},\;\phi_{12}^{2}=\frac{k_{2}^{2}}{{\left(k_{1}+k_{2}-m_{1}\omega_{n2}^{2}\right)}^{2}m_{2}+m_{1}k_{2}^{2}}$$

and

A 7*b* $$\phi_{21}^{2}=\frac{{\left(k_{1}+k_{2}-m_{1}\omega_{n1}^{2}\right)}^{2}}{{\left(k_{1}+k_{2}-m_{1}\omega_{n1}^{2}\right)}^{2}m_{2}+m_{1}k_{2}^{2}},\;\phi_{22}^{2}=\frac{{\left(k_{1}+k_{2}-m_{1}\omega_{n2}^{2}\right)}^{2}}{{\left(k_{1}+k_{2}-m_{1}\omega_{n2}^{2}\right)}^{2}m_{2}+m_{1}k_{2}^{2}}.$$

For the system shown in figure 5 with *Ψ*_1_ = 0 and *Ψ*_2_ = 0, it satisfies parameter conditions described in equations (3.6) and (3.7). The modeshapes in expressions (A 7) can be further simplified as

A 8*a* $$\phi_{11}^{2}=\frac{1}{2m_{1}\left(1+\mu +\sqrt{(1+\mu )/\mu }\mu \right)},\;\phi_{12}^{2}=\frac{1}{2m_{1}\left(1+\mu -\sqrt{(1+\mu )/\mu }\mu \right)}$$

and

A 8*b* $$\phi_{21}^{2}=\frac{{\left(\sqrt{(1+\mu )/\mu }+1\right)}^{2}}{2m_{1}\left(1+\mu +\sqrt{(1+\mu )/\mu }\mu \right)},\;\phi_{22}^{2}=\frac{{\left(\sqrt{(1+\mu )/\mu }-1\right)}^{2}}{2m_{1}\left(1+\mu -\sqrt{(1+\mu )/\mu }\mu \right)}.$$

where *μ* = m_1_/m_2_, which is the mass ratio.

For the specific system, shown schematically in figure 1, with symmetric linear parameters, i.e. m_1_ = m_2_ and k_1_ = k_3_, the modeshape elements satisfy *ϕ*_11_ = *ϕ*_12_ = *ϕ*_21_ = − *ϕ*_22_, obtained from expressions (A 7). Thus, *Ψ*_1_ and *Ψ*_2_, expressed in equations (A 5), can be reduced to

A 9 $$\Psi_{1}=\Psi_{2}=\phi_{11}^{4}\left(\alpha_{1}-\alpha_{3}\right).$$

As discussed in §2, obtaining dynamic symmetry requires two single-mode solutions, i.e. satisfying *Ψ*_1_ = 0 and *Ψ*_2_ = 0. As seen from equations (A 9), *Ψ*_1_ = 0 and *Ψ*_2_ = 0 can only be satisfied when *α*_1_ = *α*_3_, which means having symmetric nonlinear parameters. In summary, to obtain dynamic symmetry for a system with linear symmetry requires nonlinear symmetry.

## Appendix B. List of coefficients

B 1 $$\begin{array}{rl} g_{3} & =-19683{\left(\phi_{11}^{2}+\phi_{12}^{2}\right)}^{4}{\left(\phi_{11}\delta_{11}+\phi_{12}\delta_{12}\right)}^{2}{\left(\phi_{11}\delta_{12}-\phi_{12}\delta_{11}\right)}^{6}, \end{array}$$

B 2 $$\begin{array}{rl} g_{5} & =-19683{\left(\delta_{11}^{2}+\delta_{12}^{2}\right)}^{4}{\left(\phi_{11}\delta_{11}+\phi_{12}\delta_{12}\right)}^{2}{\left(\phi_{11}\delta_{12}-\phi_{12}\delta_{11}\right)}^{6} \end{array}$$

B 3 $$\begin{array}{rl} \;\mathrm{and}\;g_{4} & =-19683P{\left(\phi_{11}\delta_{12}-\phi_{12}\delta_{11}\right)}^{6}, \end{array}$$

where

B 4 $$\begin{array}{rl} P & =\left(2\phi_{11}^{6}-8\phi_{11}^{4}\phi_{12}^{2}-\frac{2}{3}\phi_{11}^{2}\phi_{12}^{4}-\frac{4}{27}\phi_{12}^{6}\right)\delta_{11}^{6}+\left(2\phi_{12}^{6}-8\phi_{11}^{2}\phi_{12}^{4}-\frac{2}{3}\phi_{11}^{4}\phi_{12}^{2}-\frac{4}{27}\phi_{11}^{6}\right)\delta_{12}^{6}\\ & \;+\left(28\phi_{11}^{5}\phi_{12}-\frac{88}{3}\phi_{11}^{3}\phi_{12}^{3}-\frac{4}{9}\phi_{11}\phi_{12}^{5}\right)\delta_{11}^{5}\delta_{12}+\left(28\phi_{11}\phi_{12}^{5}-\frac{88}{3}\phi_{11}^{3}\phi_{12}^{3}-\frac{4}{9}\phi_{11}^{5}\phi_{12}\right)\delta_{11}\delta_{12}^{5}\\ & \;+\left(-8\phi_{11}^{6}+90\phi_{11}^{4}\phi_{12}^{2}-\frac{404}{9}\phi_{11}^{2}\phi_{12}^{4}-\frac{2}{3}\phi_{12}^{6}\right)\delta_{11}^{4}\delta_{12}^{2}\\ & \;+\left(-8\phi_{12}^{6}+90\phi_{11}^{2}\phi_{12}^{4}-\frac{404}{9}\phi_{11}^{4}\phi_{12}^{2}-\frac{2}{3}\phi_{11}^{6}\right)\delta_{11}^{2}\delta_{12}^{4}\\ & \;+\left(-\frac{88}{3}\phi_{11}^{5}\phi_{12}+\frac{3536}{27}\phi_{11}^{3}\phi_{12}^{3}-\frac{88}{3}\phi_{12}^{5}\phi_{11}\right)\delta_{11}^{3}\delta_{12}^{3}, \end{array}$$

and where *δ*_11_ = *ϕ*_11_ − *ϕ*_21_ and *δ*_12_ = *ϕ*_12_ − *ϕ*_22_.
